# Brain Structural Alterations Underlying Mood-Related Deficits in Schizophrenia

**DOI:** 10.3390/biomedicines13030736

**Published:** 2025-03-18

**Authors:** Margherita Biondi, Marco Marino, Dante Mantini, Chiara Spironelli

**Affiliations:** 1 Padova Neuroscience Center, University of Padova, 35131 Padova, Italy; margherita.biondi.1@phd.unipd.it; 2 Department of General Psychology, University of Padova, 35131 Padova, Italy; marco.marino@unipd.it; 3 Movement Control and Neuroplasticity Research Group, KU Leuven, 3001 Leuven, Belgium; dante.mantini@kuleuven.be

**Keywords:** voxel-based morphometry (VBM), structural MRI (sMRI), schizophrenia, mood symptoms

## Abstract

**Background/Objectives:** Schizophrenia (SZ) is a complex psychiatric disorder characterized by neurodegenerative processes, but the structural brain alterations associated with its progression remain poorly understood. This study investigated structural brain changes in SZ, particularly in the fronto-temporal and limbic regions, and explored their relationship with symptom severity, with a focus on mood- and emotion-related symptoms. **Methods:** We analyzed structural MRI data from 74 SZ patients and 91 healthy controls (HCs) using voxel-based morphometry (VBM) to compare whole-brain grey matter volumes (GMVs). The analysis focused on the fronto-temporal and limbic regions, and correlations between GMV and symptom severity were assessed using the Positive and Negative Syndrome Scale (PANSS) and the Generalized Psychopathology (GP) scale. **Results:** SZ patients exhibited significant reductions in GMV in the fronto-temporal and limbic regions, including the dorsolateral prefrontal cortex (dlPFC) and the temporal pole, compared to HCs. Notably, a significant positive association was found between GMV in the right inferior temporal gyrus (ITG) and the severity of generalized psychopathology, as well as with anxiety, depression, mannerisms, and unusual thought content. Further post hoc analysis identified a specific cluster of mood-related symptoms contributing to the GP scale, which correlated with GMV changes in the right ITG. **Conclusions:** Our findings provide new evidence of structural brain alterations in SZ, particularly in the fronto-temporal and limbic regions, suggesting a progressive neurodegenerative pattern. The role of the right ITG in mood- and emotion-related symptoms requires further exploration, as it could offer insights into SZ pathophysiology and aid in distinguishing SZ from other mood-related disorders.

## 1. Introduction

Schizophrenia (SZ) is a severe and chronic psychiatric disorder, with an estimated prevalence of 0.45% among the global adult population [1]. The syndromic profile comprises a set of complex and heterogeneous symptoms, classifiable as disturbances of thought (e.g., delusions), behavior (e.g., grossly disorganized motricity), cognition (e.g., attentional deficits), and emotions (e.g., flat affect) [2]. Moreover, multidimensionality is a key aspect of the etiopathological hypotheses for SZ onset and course: past studies have revealed an interplay between biological (e.g., genetic variations, neurotransmitter dysfunctions), socioenvironmental (e.g., urban residence, substance use), and psychological (e.g., childhood trauma, stress) factors in determining vulnerability to and persistence of SZ [3]. However, despite significant progress in understanding this disorder, no definitive etiological and pathophysiological factors have been identified to date [3].

Among the candidate correlates, several neurodevelopmental abnormalities have been consistently traced in SZ patients, such as genetic predispositions, pre- and peri-natal complications, minor physical abnormalities, and early neurocognitive dysfunction [4]. These developmental disorders are believed to predispose individuals to SZ, the onset of which requires further interaction with external (e.g., social adversity) and internal (e.g., stress) triggers [5]. The neurodevelopmental model is dominant among SZ etiopathological theories, but it is not exhaustive: it does not account for the relentless decline that characterizes the chronic course of SZ [6]. As such, researchers have more recently begun focusing on alternative, though not exclusionary, mechanisms of neurodegeneration, i.e., the progressive loss of neuronal structural and functional features [7]. Both post mortem observations of neurochemical alterations (e.g., excitotoxicity) and in vivo evidence of brain alterations (e.g., cortical volume loss) support this hypothesis [8]. In particular, the occurrence of progressive abnormalities in cerebral morphology has been suggested as a macroscale marker of neurodegeneration in SZ [8].

Considerable reductions in the size of various brain areas have been repeatedly found in individuals with SZ, mainly reflecting a loss of grey matter volume (GMV) and an increase in ventricle size in several cortical and subcortical regions (e.g., [9,10,11]). The most pronounced GMV losses have been consistently identified in the frontal and temporal lobes [11]. Furthermore, Howes and colleagues (2023), through a meta-analysis that included more than 600 studies and 23,000 patients, showed that GMV was reduced in patients with chronic SZ, in those experiencing their first episode of SZ, and, to a lesser extent, in individuals with risk factors for SZ [11]. This suggests that the degenerative process could begin during the prodromal phase of the disorder and persist throughout its course. Relevant supporting evidence stems from a series of longitudinal imaging studies (e.g., [12,13,14]). Among these, Andreasen and colleagues (2011) analyzed the scans of 202 patients followed up to 15 years, concluding that the neuroprogressive component (i.e., a faster tissue volume decrement in patients compared to controls) is present in SZ from the first episode [15].

While the debate on whether SZ should be conceptualized solely as a neurodevelopmental or a neurodegenerative disorder is still ongoing, the idea of a non-exclusive model merging both types of evidence is gaining consensus (e.g., [16,17,18]). These processes can be compatible and coexistent by considering that SZ may involve a dynamic interaction between early neurodevelopmental abnormalities and later neurodegenerative impairments [18]. Regarding the latter, voxel-based morphometry (VBM) analysis—an advanced and fully automated technique based on structural magnetic resonance imaging (MRI) data—enables voxel-by-voxel comparisons of GMV in patients to healthy controls in a noninvasive, rapid, and unbiased manner [19]. This method has proven essential for pinpointing the presence of brain morphological changes (e.g., [20,21,22]) and is crucial for monitoring the evolution of neurodegeneration indices in SZ. VBM data have been progressively integrated into various voxel-wise coordinate-based meta-analyses to systematically examine the structural alterations occurring in SZ (e.g., [21,23,24,25,26,27,28,29,30]), including studies of high-risk individuals, first-episode patients, and chronic patients [24], as well as early-onset patients [30], and studies focusing on the influence of factors like gender [26] and drug use [27,28]. Notably, some important findings have emerged consistently across different SZ conditions, including GMV reductions mainly in the fronto-temporal lobe, and secondarily in the cingulate cortex and the insula [21,23,24,25,26,27,28,29,30].

Finally, an equally important aspect to investigate is the relationship between SZ neural correlates and its symptomatology. While a discrete body of literature exists on the distinctive psychotic symptoms of SZ (e.g., [31,32,33]), and cognitive deficits are believed to primarily depend on neurodevelopmental alterations—since they often arise before the illness onset [34,35]— how emotion- and mood-related symptoms can inform us about the nature and progression of SZ remains largely unclear. However, emotions and mood are significantly altered in SZ patients, often manifesting as diminished emotional range, sense of purpose, interest, or social drive [36], along with depressive and anxious manifestations [37] and emotional dysregulation [38]. Investigating the association between these symptoms and the neural underpinnings of neurodegeneration could elucidate important pathophysiological aspects of SZ, offering, for example, insights into the definition of different phenotypes of this disorder.

In this study, we combined neuroimaging data and symptomatology to investigate the neural correlates of psychopathology in SZ, with a special focus on items related to emotion and mood. Specifically, we implemented a VBM approach to explore the brain structural alterations associated with SZ and examined their relationship with symptom severity. In agreement with past literature [11,21,23,24,25,26,27,28,29,30], we expected to find GMV reductions in the fronto-temporal region in SZ patients compared to healthy controls as a possible marker of neurodegeneration, and to identify correlations of neural alterations in the limbic system—an ensemble of key regions for emotional and mood regulation [39]—with items primarily associated with emotion- and mood-related symptom dimensions.

## 2. Materials and Methods

### 2.1. Participants

Structural MRI (sMRI) data from SZ patients and healthy controls (HCs) were obtained from COINS (https://coins.trendscenter.org), an online, open-access database designed for research purposes that integrates data from multiple sources, including the Center for Biomedical Research Excellence (COBRE), MCIC (via the COINS database), the Functional Biomedical Informatics Research Network (FBIRN), XNAT Central, and the NUNDA and REDCap frameworks from Northwestern University. By providing access to these heterogeneous databases, COINS enables large-scale studies based on multi-site, multi-dimensional, and multi-modal data. We outlined the following criteria in a query on COINS: (1) availability of T1 sMRI data and (2) availability of complete Positive and Negative Syndrome Scale (PANSS) scores [40] (for SZ patients only). The resulting sample was derived from the COBRE project, which collects data from psychiatric patients at the University of New Mexico (UNM) Psychiatric Center, the Raymond G. Murphy Veterans Affairs Medical Center, and other clinics within the Albuquerque metropolitan area (USA); healthy individuals were also recruited in Albuquerque. According to the COBRE guidelines, SZ patients had to meet specific inclusion criteria: (1) a diagnosis of SZ confirmed by two psychiatrists using the DSM-IV Structured Clinical Interview for Axis I disorders (SCID) [41]; (2) clinical stability, assessed 3 months prior to and during the MRI sessions; and (3) an age range of 18–65 years. Clinical data about the patients were also available, including age of onset, illness duration, and medication (details in Table 1). The criteria for HC selection were based on the SCID-Non-Patient administration: (1) no diagnosis of any current or past Axis I psychiatric disorder; (2) no history of head trauma (with loss of consciousness greater than 5 min); (3) no substance abuse, addiction, or antidepressant use in the past 5 months; and (4) absence of psychotic disorders among first-degree relatives.

COBRE-funded data collection took place at the Mind Research Network, subject to the licensing procedure 5P20RR021938/P20GM103472 from the National Institutes of Health (NIH) to Dr. Vince Calhoun. Data were downloaded via the Collaborative Informatics and Neuroimaging Suite Data Exchange (COINS; http://coins.trendscenter.org). All participants in the COBRE project provided written informed consent, and their data were anonymized prior to access to ensure privacy. Additionally, structural images were visually inspected to confirm their suitability.

### 2.2. MRI Data Acquisition

MRI data were obtained using a 3T Siemens MR scanner (Trio, Siemens Healthcare, Erlangen, Germany). The acquisition protocol involved sagittal gradient echo-scout images through the midline, with image slices taken in axial, oblique, and parallel orientations to the antero-posterior commissure (AC-PC) line; oblique slices were used to minimize the orbitofrontal susceptibility artifact. High-resolution T1-weighted images were acquired with a multi-echo MP-RAGE sequence (five echoes) with the following parameters: TE (Echo Times) = 1.64, 3.5, 5.36, 7.22, 9.08 ms, TR (Repetition Time) = 2.53 s, TI (Inversion Time) = 1.2 s, flip angle = 7°, Number of Excitations (NEX) = 1, slice thickness = 1 mm, FOV (Field of View) = 256 mm, resolution = 256 × 256. The first image of each slot was removed to account for T1 equilibrium effects. The scanning parameters can be found at the COINS website.

### 2.3. Whole-Brain VBM Analysis

To conduct a voxel-wise estimation of the GMV [42], VBM analysis [19] was performed using Statistical Parametric Mapping 12 (SPM12) software (https://www.fil.ion.ucl.ac.uk/spm/software/spm12/) in the MATLAB^®^ (MathWorks Inc., Natick, MA, USA) environment. This analysis was conducted on each individual structural MRI using the Computational Anatomy Toolbox (CAT) extension to SPM12 (http://www.neuro.uni-jena.de/cat/) [43], which includes diverse morphometric methods.

All T1-weighted images were preprocessed using the following steps: (1) spatial registration to the Montreal Neurological Institute (MNI) brain template, (2) tissue segmentation into grey matter, white matter, and cerebrospinal fluid, and (3) bias correction of intensity non-uniformities. Finally, the segmented grey matter images were modulated by scaling them with the amount of volume changes due to spatial registration, resulting in modulated grey matter images used for statistical analysis [44,45]. A two-tailed two-sample t-test was employed to test the difference in GMV between the HC and SZ patient groups. The False Discovery Rate (FDR) correction was used to correct for multiple comparisons.

In addition, to test the contribution of relevant clinical variables in the SZ group, we performed regression analyses between whole-brain VBM GMV values and age of onset, illness duration, and medication.

### 2.4. Regression Analysis Focused on VBM-GMV and Symptom Severity

We investigated the association between regional GMV and PANSS symptom severity indices in SZ patients. PANSS includes three different symptom severity scores, calculated by summing the ratings for the items within each domain: (1) Positive Scale total score (i.e., the overall measure of the severity of positive symptoms, such as delusions and hallucinations); (2) Negative Scale total score (i.e., the overall measure of the severity of negative symptoms, such as blunted affect and emotional withdrawal); and (3) Generalized Psychopathology Scale total score (i.e., the overall measure of the severity of general psychiatric symptoms, such as mood and cognitive impairments) [40]. The FDR correction was used to correct for multiple comparisons.

## 3. Results

### 3.1. Demographics and Clinical Data

The final sample consisted of 74 SZ patients and 91 HCs, similar in age and gender distribution (Table 1). The clinical characteristics of patients are summarized in Table 1 (mean ± standard deviations): on average, the patients had an onset age of 22.61 ± 8.85 and an illness duration of 14.82 ± 12.33 years; all patients were medicated, and drug treatments were calculated as an equivalent dose of olanzapine and chlorpromazine. We conducted an additional analysis within the SZ sample to check for clinical differences between male (SZ-*m*, n = 60) and female (SZ-*f*, n = 14) patients, and no significant differences emerged (see Table 1).

### 3.2. Whole-Brain VBM Analysis

**Figure 1:**
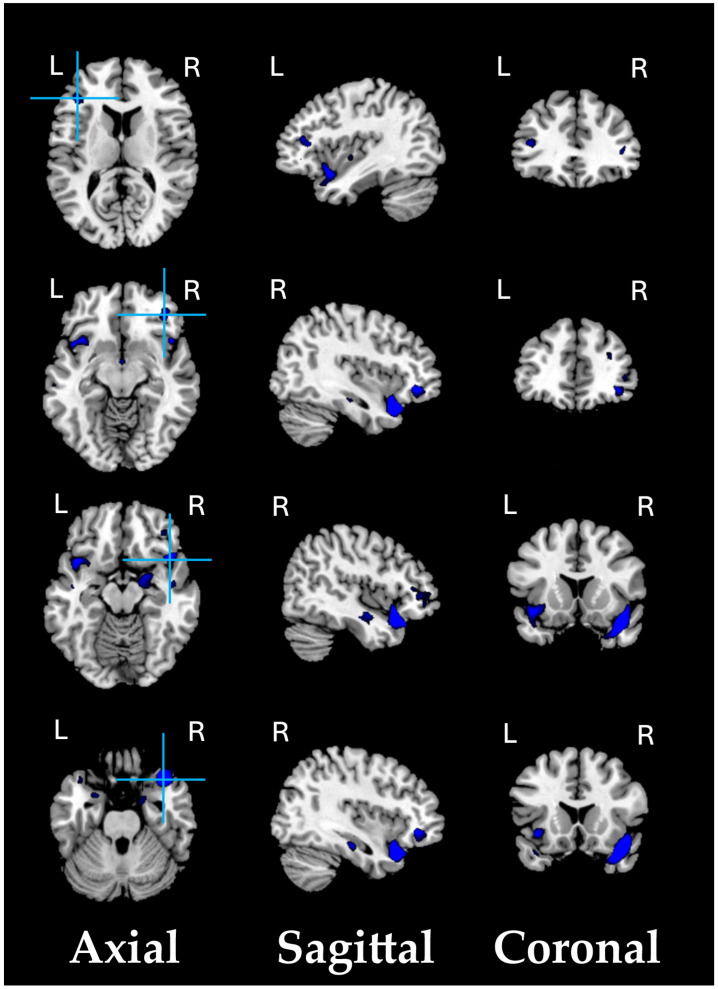
Significant GMV atrophic regions in SZ patients compared to HCs (*q* < 0.001).

As a preliminary analysis, we conducted a structural VBM-GMV analysis on 74 SZ patients and 91 healthy controls. No significant effects of clinical covariates (i.e., age of onset, illness duration, and medication) on the whole-brain VBM-GMV values in SZ patients were found. A significant between-group difference (HC > SZ, i.e., GMV atrophy in patients) was observed, with t = 4.39, *p* = 0.00001 (FDR-corrected *q* < 0.001), Cohen’s d = 0.688. Figure 1 shows the significant GMV atrophy found in the brains of SZ patients, and Table 2 lists the corresponding MNI coordinates.

### 3.3. Regression Analysis Focused on VBM-GMV and Symptom Severity

Regression analysis was conducted to examine the relationship between regional GMV values and symptom severity in SZ patients. This analysis considered the PANSS Positive Scale, Negative Scale, and Generalized Psychopathology scale. On average, SZ patients scored as follows (mean ± standard deviation): (1) Positive Scale total score = 14.99 ± 4.86; (2) Negative Scale total score = 15.15 ± 5.49; (3) Generalized Psychopathology scale total score = 28.57 ± 8.77.

**Figure 2:**
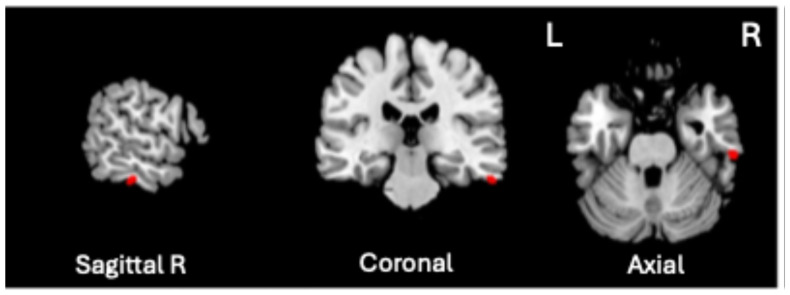
Significant positive association between the VBM-GMV in the right inferior temporal gyrus (ITG) and the severity of Generalized Psychopathology PANSS subscale in SZ patients (*q* < 0.01).

A significant positive association was found between VBM-GMV and Generalized Psychopathology scores only (t = 4.72, *p* = 0.0000057, *q* < 0.01, volume size = 82 mm^3^; Cohen’s d = 1.112). As shown in Figure 2, the greater the severity of Generalized Psychopathology, the greater the GMV in the right inferior temporal gyrus (ITG), corresponding to Brodmann Area (BA) 20 (MNI coordinates 61, −26, −28).

### 3.4. Post Hoc Analysis of PANSS Scores: Generalized Psychopathology (GP) Items

Since the Generalized Psychopathology scale includes a variety of additional SZ symptoms, we performed a post hoc analysis to further clarify the potential role of specific symptom clusters within the main scale. We conducted a Pearson correlation analysis between the Generalized Psychopathology scale scores and each item of this scale. As shown in Table 3, significant positive correlations (*q* < 0.001) were found with nine items, primarily associated with mood-related symptom dimensions (i.e., GP02—Anxiety, GP03—Guilt feelings, GP04—Tension, GP05—Mannerisms and posturing, GP06—Depression, GP09—Unusual thought content, GP13—Disturbance of volition, GP15—Preoccupation, GP16—Active social avoidance).

### 3.5. Post Hoc Analysis of ROI-GMV and Selected GP Items

**Figure 3:**
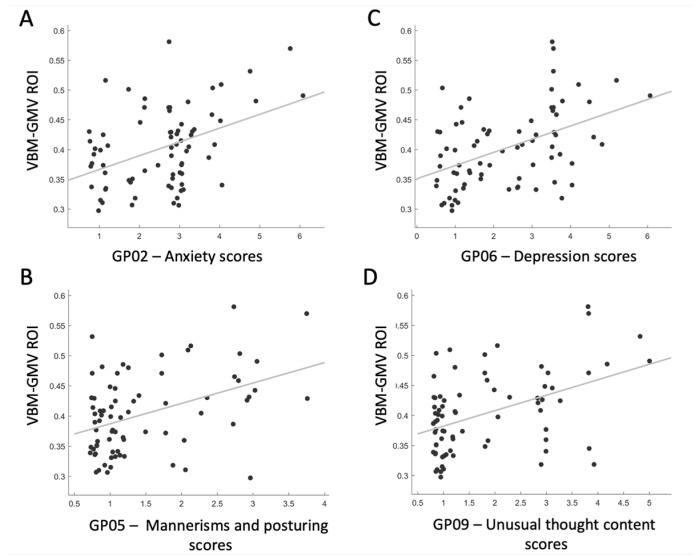
Correlation plots showing significant relationships between the VBM-GMV in the right inferior temporal gyrus (ITG) and the items contributing the most to the Generalized Psychopathology PANSS subscale in SZ patients (*q* < 0.01).

As the post hoc analysis of PANSS scores for GP items revealed that a specific cluster of mood-related symptoms primarily contributed to the GP scale, we decided to further investigate the association between this cluster of symptoms and the VBM-GMV, which was significantly associated with the severity of the GP scale. We defined a region of interest (ROI) centered on the right ITG, specified as a 6 mm radius sphere (BA 20; MNI coordinates: 61, −26, −28). Four significant positive associations were found between the ROI and the following: GP02—Anxiety scores (t = 3.89, *p* = 0.0002, *q* < 0.01, Cohen’s d = 0.910), GP05—Mannerisms and posturing scores (t = 3.89, *p* = 0.0002, *q* < 0.01, Cohen’s d = 0.910), GP06—Depression scores (t = 4.43, *p* = 0.00003, *q* < 0.01, Cohen’s d = 1.037), and GP09—Unusual thought content scores (t = 4.24, *p* = 0.00006, *q* < 0.01, Cohen’s d = 0.992). As shown in Figure 3, the greater the VBM-GMV in the right ITG, the more severe the levels of anxiety and depression (panels A and C), the more bizarre or disorganized the movements and posture (panel B), and the more strange, fantastic, or bizarre the ideas and thought content (panel D). 

Compared with HC participants, SZ patients’ VBM-GMV at the ROI level tended to be reduced, pointing to overall atrophy of the right ITG (details in Figure S1, in the Supplementary Materials).

## 4. Discussion

Increasing evidence suggests that neurodegeneration might be a crucial mechanism in SZ, particularly in determining the progressive decline that characterizes its disease course [6,8,16,17,18]. Anomalies in brain morphology have been proposed as neurodegenerative macroscale markers [8], but despite consistent evidence of structural alterations occurring in this disorder [11,21,23,24,25,26,27,28,29,30], no definitive etiopathological correlates have been identified to date. Additionally, the association between the heterogeneous symptoms of SZ and cerebral alterations remains unclear, particularly for mood- and emotion-related symptoms, although their impact on SZ patients has been increasingly recognized [36,37,38]. In the present study, we aimed to address these questions by investigating the presence of structural alterations in SZ patients, both in comparison with HCs and in association with symptom severity. Specifically, we conducted whole-brain VBM analysis to identify morphological differences between patients and HCs, followed by testing the association between regional GMV and symptom severity indices in SZ patients, considering PANSS Positive, Negative, and Generalized Psychopathology scales.

### 4.1. GMV Reductions in SZ Patients

As a primary outcome, we found that SZ patients exhibited GMV reductions in a range of fronto-temporal and limbic regions compared to HCs. Notably, in line with our hypothesis and a consistent body of literature [11,21,23,24,25,26,27,28,29,30], our SZ group was characterized by fronto-temporal atrophy, replicating the most consistent finding of GMV loss in this disorder. In particular, the frontal lobe in SZ is affected by structural, functional, and metabolic alterations, which together suggest its involvement in the pathophysiology of the disorder [46]. Among the affected regions, we observed reduced GMV in the dorsolateral prefrontal cortex (dlPFC), a long-standing area of interest in SZ. Abnormalities in this region’s structure have been repeatedly identified in patients compared to healthy individuals (e.g., [47,48]), along with neurochemical alterations (e.g., [49,50]) and, most notably, functional impairments (e.g., [51,52,53]). The dlPFC plays a key role in various higher-order cognitive functions, primarily cognitive control [54]—also in social contexts [55]—working memory [56], and executive function [57], all of which are well-known to be impaired in SZ patients [58]. Increasing evidence links neural alterations in the dlPFC to cognitive deficits in this disorder (e.g., [59,60]). Specifically, SZ patients fail to activate the dlPFC to the same extent as healthy individuals, a phenomenon known as hypofrontality, which is believed to play a core role in SZ functioning [61]. Notably, as suggested by Smuncy and colleagues (2022) in their review, neurocognitive impairment in SZ might result from dysfunctional neuronal activity in the dlPFC (i.e., hypofrontality), plausibly linked to the neuroanatomical abnormalities observed in this region [48]. Considering the consistency of dlPFC volumetric reductions in SZ patients compared to HCs, it is important to question whether medications play a modulatory role. While previous studies have reported the effect of antipsychotic drugs on dlPFC grey matter loss (see, e.g., [62] for a review), we did not find any association between medications and GMV values in our SZ sample. Further evidence has shown that reduced GMV in the dlPFC is also present in never-medicated, first-episode SZ patients [63] and, to a lesser degree, in individuals at high risk for psychosis, as reviewed by Andreou and Borgwardt [64]. These findings suggest that these alterations might not solely be a consequence of drug treatment. Indeed, these dlPFC findings exemplify how structural anomalies might be present at illness onset or even earlier, following a progressive pattern in the chronic stage [65].

Evidence for the progression of cortical changes, particularly grey matter loss (more pronounced in chronic rather than early-phase SZ patients), is available not only for the frontal lobe but also for the temporal cortex [66]. In our study, SZ patients exhibited lower GMV in the bilateral temporal pole compared to HCs, replicating previous findings in the literature. These findings span from chronic patients [67,68] to first-episode individuals [69,70] and at-risk relatives [71]. Notably, the progressive decline in the temporal pole has been corroborated in a large meta-analysis by the ENIGMA Consortium (2018), which reported a stronger negative correlation between age and cortical thickness in the bilateral temporal pole of 4474 individuals with SZ compared to healthy volunteers [72].

Focusing on symptomatic implications, we did not find any associations between temporal pole GMV and PANSS severity scores. However, previous whole-brain VBM studies identified significant correlations between GMV reduction in the temporal pole and disrupted behaviors, such as poor theory of mind performance [73] and severe formal thought disorder [74]. These findings used assessments that may be better suited to capturing the functions in which the temporal pole is involved, such as a behavioral task to assess theory of mind skills [73] or a specific scale for assessing thought and language [74]. Indeed, the temporal pole plays an important role in language, multisensory integration, and socio-emotional processes (see, e.g., [75] for a review)—domains compromised in SZ, similar to the functions sustained by the dlPFC (as discussed above).

Overall, in line with previous literature, our results point to a consistent pattern of fronto-temporal alterations in SZ: frontal and temporal regions—and their mutual reduced connectivity shown by both functional MRI [76] and EEG [77] studies—are characterized by reduced GMV in SZ patients. Given the fundamental role of the fronto-temporal networks in cognitive control operations [78], it is plausible that structural and functional alterations in these regions might contribute to the onset of cognitive deficits, the prevalence and pervasiveness of which has been widely demonstrated in SZ [79]. This has been proven, for instance, by Alkan and colleagues (2021), who found lower performance in various cognitive domains (e.g., attention, visual learning) in SZ to be associated with fronto-temporal cortical thickness changes [80]. The fronto-temporal atrophic pattern is also evident in drug-naïve, first-episode subgroups and clinically high-risk individuals, even after accounting for confounding factors such as antipsychotic use. These findings support the interpretation that progressive fronto-temporal brain atrophy may be a hallmark of SZ [81]. Notably, SZ shares these patterns of brain atrophy with fronto-temporal dementia, a neurological condition characterized by degeneration of the frontal and temporal lobes [82,83]. Moreover, the two pathologies exhibit notable overlaps in clinical presentation, including positive and negative symptoms [82]: interestingly, neuroimaging and neuropsychological evidence has shown that, in both SZ and fronto-temporal dementia, positive symptoms are primarily linked to pathology in the temporal cortex, while negative symptoms are associated with abnormalities in the prefrontal cortex [84].

Beyond our hypothesis, we also found reduced GMV in SZ patients compared to HCs in the limbic system, specifically in the amygdala and the parahippocampus. Although this result is not novel in the SZ literature [85], structural alterations in these regions, as well as dysfunction in the prefrontal–limbic circuitry [86], have also been associated with mood-related disorders, such as major depression, bipolar disorder, and anxiety disorders [87]. The limbic system is primarily responsible for emotional behavior [39], processing emotional inputs, and regulating the expression of emotions in both healthy and pathological conditions [88]. It also contributes to cognitive processes such as memory, learning, and motivation, thanks to its complex network structure [89]. The amygdala is a central hub at the intersection of emotion and cognition [90], and is implicated in various emotional functions, from fear responses to positive-valence emotions regulation and reward mechanisms [91], as well as memory, learning, perception, and attention [92]. The parahippocampus also plays a critical role where emotion and cognition intersect, with particular specialization in episodic memory and information processing [93]. Consistent with their functional roles, structural alterations in both these regions have been primarily associated with major depression, as shown by a recent meta-analysis (2024) [94]. In line with this, we hypothesized that neural changes in the limbic system might underlie emotion- and mood-related symptoms in SZ. However, we did not find any significant correlation between GMV values in limbic areas and severity scores of the Positive, Negative, and Generalized Psychopathology scales. It is plausible that limbic alterations play a role in determining and maintaining affective symptoms in a pathognomonic way for mood disorders, as demonstrated by a long-standing body of literature (see, e.g., [95] for a review), while this link may not be as straightforward in SZ. Indeed, according to the Diagnostic and Statistical Manual of Mental Disorders (DSM-5), clinicians should carefully differentiate between SZ and mood disorders. A diagnosis of schizoaffective disorder should be made only if a major depressive or manic episode occurs simultaneously with the psychotic active phase, and mood symptoms are present for most of the duration of active periods [2]. Therefore, different brain areas may underlie emotion- and mood-related symptoms in these disorders. However, going beyond the thresholds for overt symptom classification, the role of the limbic system in emotion regulation is also crucial among individuals with SZ: for instance, a meta-analysis (2019) of functional neuroimaging studies examining a total of 474 individuals with SZ vs. 472 HCs found that SZ patients exhibited increased activation in several limbic regions in response to neutral stimuli, suggesting aberrant attribution of emotional significance to non-threatening inputs [96]. Nevertheless, our study showed GMV reduction in the amygdala and parahippocampus regions within the limbic system, a result that is consistent with previous voxel-wise coordinate-based meta-analyses [23,24,25] and adds new evidence regarding the atrophic pattern in SZ patients. Notably, a progressive decline was also observed in these areas: in a longitudinal study, individuals at ultra-high risk of developing psychosis showed GMV reduction in the parahippocampus when they developed psychosis, as confirmed by MRI scans after at least 12 months [97]. A more prominent decrease in amygdala density was found in older patients than in younger patients in a study by Pol et al. (2001) [98]. These authors supported the hypothesis that neurodegeneration may contribute to SZ, in addition to neurodevelopmental processes [98].

We conclude that the loss of GMV in limbic and fronto-temporal regions found in SZ patients compared to HCs aligns with the widespread atrophic results in the literature, which altogether suggest the occurrence of pathomorphological progression in this disorder, from at-risk individuals to chronic patients [99]. According to many authors, these findings are in support of the presence of a neurodegenerative process in SZ, as it can explain key features of chronic SZ, which are mainly associated with the heterogeneous but commonly deteriorating course of the illness [100]. Indeed, while brain structural alterations could also be attributed to altered neurodevelopment, altered neurodevelopment cannot explain the progressive course of GMV atrophy found in SZ patients which is reported for fronto-temporal and limbic regions, as shown by the studies discussed above (e.g., [65,66,81,97,98]). According to Pérez-Neri and colleagues (2006), this is instead indicative of an active neurodegenerative process, which, however, does not exclude the relevant neurodevelopmental abnormalities of SZ [8]. For instance, the authors pointed out that both neuronal loss (i.e., neurodegeneration) and altered development of the central nervous system may result from reduced neurotrophic factor signaling [8]. In line with this integrated hypothesis, Vita and colleagues (2012) found significantly higher GMV losses over time in 813 SZ patients compared to 718 HCs in their meta-analysis of longitudinal MRI studies, and they proposed that this finding could be interpreted as the existence of a cortical neurodegenerative process acting differently at different disease stages [13]; at the same time, they did not exclude the influence of early neurodevelopmental abnormalities, suggesting a plausible interaction between anomalous neurodevelopmental and neurodegenerative processes leading to accelerated tissue loss [13]. This perspective also aligns with Stone et al. (2022), who proposed a revisited model for SZ that combines neurodevelopmental influences in early life with neurodegenerative processes in later life, such as the decline in biological integrity [6]. In conclusion, SZ might occur with neurodegenerative processes, explaining the decline of its chronic course, that are superimposed on pre-existing neurodevelopmental abnormalities [101].

### 4.2. Correlation Between GMV Alteration and Generalized Psychopathology Scores in SZ Patients

Correlation analysis between whole-brain VBM-GMV values in SZ patients and PANSS symptom severity scores revealed a significant positive association between right ITG GMV and the Generalized Psychopathology scale total score: the greater the GMV in this region, the greater the severity of Generalized Psychopathology. However, the GP scale focuses on a broader range of symptoms that do not fall under the positive or negative categories, measuring aspects related to both mood and cognition. Given this heterogeneity, we examined the role of specific symptom clusters in the main scale, correlating the GP total scores with each item of the scale. We found that nine items contributed the most, all related to the mood dimension. Among these items, four positively correlated with the ROI that we set post hoc in the right ITG: the greater the GMV in the right ITG, the more severe the levels of anxiety and depression, the more bizarre or disorganized the movements and posture, and the more atypical the ideas.

Some research indicates that, among the multiple high-level cognitive functions in which the ITG is involved (e.g., visual recognition, decision making, language), it also plays a role in emotional processing and regulation [102]. For example, Su and colleagues (2015) found that patients with somatization disorder (i.e., a disorder characterized by multiple somatic complaints accompanied by distress and psychosocial disability [103]) presented with increased functional connectivity strength in the right ITG compared to HCs, and that connectivity values in this region positively correlated with the severity of anxiety [104]. Moreover, Dung et al. (2016) found that, in healthy individuals, more severe early-life stress, especially emotional neglect, corresponded to higher activity in the right ITG [105]. Interestingly, an opposite pattern emerged in the case of alexithymia, which is the inability to express, describe, or distinguish one’s emotions [106]. As demonstrated by the study by Deng et al. (2013), high-alexithymic females had significantly less activation in the right ITG compared to low-alexithymic females in response to negative, high-arousal emotional pictures [107]. Altogether, these findings suggest the functional involvement of the right ITG in emotional processes, which may also result in structural effects in psychopathology. For example, a voxel-wise coordinate-based meta-analysis of social anxiety disorder (2018) showed that patients exhibited greater right ITG volume compared to healthy controls [108], in agreement with the results from our study, where greater GMV in the right ITG was associated with symptoms such as anxiety and depression. For this reason, we propose that our findings in SZ patients may depend on the type of symptoms to which they are linked, specifically emotion- and mood-related symptoms. Indeed, when compared with HC participants, the VBM-GMV in SZ patients at the ROI level tended to be reduced (see Supplementary Materials). Although most VBM studies found no significant grey matter changes in this region in SZ [109,110]—and, in agreement with past literature, this result did not arise from our whole-brain analysis—two previous studies found a bilateral reduction of the ITG in both first-episode [111] and chronic SZ patients [112]. Hence, a smaller ITG, albeit not a clear marker for SZ, may follow a pattern of progressive reduction along the disease course when present, aligning with the overall atrophic findings of our research.

## 5. Strengths and Limitations

Our findings strengthen the evidence for structural brain abnormalities in SZ and provide new insights into their potential clinical and functional implications, particularly in relation to mood symptoms.

First, our study confirms that SZ patients exhibit GMV reductions in fronto-temporal and limbic regions compared to HCs, using a whole-brain, unbiased approach. These alterations have been observed by previous studies across different stages of the illness, from at-risk individuals to chronic patients, supporting the idea of a progressive trajectory. In this context, our findings support the hypothesis that SZ is associated with progressive GMV loss, a hallmark of neurodegeneration that may explain the disorder’s chronic course, paralleling the neurodevelopmental framework. However, while the atrophic pattern observed in our sample is consistent with the hypothesis that atrophy is a macroscale index of neurodegeneration in SZ, the cross-sectional design of our study limited the possibility of directly addressing this interpretation. Future longitudinal studies or age-stratified analyses are needed to clarify the nature of the brain structural changes in SZ.

Second, a novel contribution of our work relates to the finding that increased right ITG GMV correlates with higher severity of generalized psychopathology symptoms, particularly those related to mood and emotion. This result aligns with prior research implicating the ITG in emotional processing and regulation, further suggesting a role for this region in affective dysregulation in SZ. Further research with larger sample sizes is required to replicate and confirm this finding, ensuring its robustness.

Finally, in the present study we also proposed a new approach for investigating the complexity of SZ symptoms, starting from a general syndromic picture and gradually increasing the focus on specific symptoms. Due to the limited size of our sample, our findings should be interpreted with caution and may not be generalizable to a larger population. However, we hope that in the future other researchers will apply the same method of analysis proposed here, as we believe this approach is particularly capable of capturing the heterogeneous distribution of SZ symptoms and their complex interaction with altered neural patterns.

## 6. Conclusions

In conclusion, we provide new and important evidence of structural brain alterations characterizing SZ patients, particularly in the fronto-temporal and limbic regions. Consistent with several prior studies, our findings suggest the presence of an atrophic pattern linked to SZ, which could serve as a macroscale marker of neurodegeneration. Future studies could build on these results by conducting longitudinal analyses to track the progression of changes in these regions over time, possibly integrating neuroimaging and molecular studies to uncover causal pathways. Finally, based on our novel finding that greater GMV in the right ITG is specifically linked to emotion- and mood-related symptoms in SZ, we suggest that the role of this region should be further explored. This could have clinical utility, for example, in differentiating SZ from other disorders, with a special focus on mood-related disorders.

## Figures and Tables

**Table 1:** Demographic and clinical characteristics of the studied sample. Mean ± standard deviations (SD) of age, onset age, illness duration, and pharmacological treatment were considered.

	SZ (n = 74)		HC (n = 91)	Statistics
Age	37.43 ± 14.02		38.54 ± 11.67	*t*_163_ = −0.55, *n.s.*
Sex (M/F)	60/14		65/26	*χ*^2^ = 1.51, *n.s.*
Age of onset	22.61 ± 8.85		*-*	
	SZ-*m*	SZ-*f*		
	22.38 ± 9.07	23.57 ± 8.04		*t*_72_ = −0.45, *n.s.*
Illness duration (years)	14.82 ± 12.33		*-*	
	SZ-*m*	SZ-*f*		
	14.78 ± 12.73	15 ± 10.86		*t*_72_ = −0.06, *n.s*
Total olanzapine equivalent dose (mg)	14.72 ± 10.46		*-*	
	SZ-*m*	SZ-*f*		
	13.7 ± 8.7	18.95 ± 15.57		*t*_72_ = 0.66, *n.s*
Total chlorpromazine equivalent dose (mg)	379.06 ± 300.78		*-*	
	SZ-*m*	SZ-*f*		
	355.97 ± 240.41	476.36 ± 480.19		*t*_72_ = −1.27, *n.s*

**Table 2:** Compared with HC adults, SZ patients exhibited statistically significant (*q* < 0.001) lower VBM-GMV (i.e., atrophy) in the fronto-temporal and limbic regions.

MNI Coordinates	Volume (mm^3^)	t-Score	Cohen’s d Value	BA	Anatomical Label
42, 19, −22	1015	6.57	1.029	38	Right-Temporalpole
39, 19, −26	834	6.33	0.992	38	Right-Temporalpole
−41, 13, −20	313	5.34	0.836	38	Left-Temporalpole
−37, 19, −27	148	5.12	0.802	38	Left-Temporalpole
−24, 4, −28	246	5.43	0.851	36	Left-Parahipp
39, 40, −10	150	5.38	0.843	47	Right-ParsOrbitalis
−39, 36, 14	142	5.20	0.814	46	Left-dlPFC
20, −1, −20	130	5.65	0.885	n/a	Right-Amygdala
23, −1, −18	115	5.85	0.916	n/a	Right-Amygdala
48, −8, 15	45	5.26	0.824	1	Right-PrimSensory

**Table 3:** Pearson‘s correlation analysis between the Generalized Psychopathology (GP) score and each item of the GP scale. (*) significant *q*-values.

GP Items	GP Scores	Exact *q*-Value	Cohen’s d Value
GP01—Somatic concern	*r* = 0.37	0.001314	0.797
GP02—Anxiety	*r* = 0.70	0.000000 (*)	1.960
GP03—Guilt feeling	*r* = 0.55	0.000000 (*)	1.317
GP04—Tension	*r* = 0.69	0.000000 (*)	1.907
GP05—Mannerisms and posturing	*r* = 0.58	0.000000 (*)	1.424
GP06—Depression	*r* = 0.72	0.000000 (*)	2.075
GP07—Motor retardation	*r* = 0.38	0.001132	0.822
GP08—Uncooperativeness	*r* = 0.29	0.01270	0.606
GP09—Unusual thought content	*r* = 0.64	0.000000 (*)	1.666
GP10—Disorientation	*r* = 0.35	0.002432	0.747
GP11—Poor attention	*r* = 0.37	0.001314	0.797
GP12—Lack of judgment and insight	*r* = 0.38	0.001132	0.822
GP13—Disturbance of volition	*r* = 0.66	0.000000 (*)	1.759
GP14—Poor impulse control	*r* = 0.41	0.000448	0.899
GP15—Preoccupation	*r* = 0.54	0.000001 (*)	1.283
GP16—Active social avoidance	*r* = 0.54	0.000001 (*)	1.283

## Data Availability

The raw data supporting the conclusions of this article were down- loaded from COINS (https://coins.trendscenter.org), a public and virtual database for research purposes.

## Supplementary Materials

The following supporting information can be downloaded at: https://www.mdpi.com/article/10.3390/biomedicines13030736/s1, Figure S1. The ROI-based GMV post hoc analysis revealed that, compared with 91 HC participants, 74 SZ patients’ VBM-GMV at the ROI level tended to be reduced (*p* < 0.01 FDR-corrected).
