# Supplementary material for: Brain Structural Alterations Underlying Mood-Related Deficits in Schizophrenia
Source: Biomedicines. 2025 Mar 18;13(3):736. doi: 10.3390/biomedicines13030736 (PMC11939877; doi:10.3390/biomedicines13030736)
Supplement: Supplementary file 1 [file biomedicines-13-00736-s001.zip › biomedicines-3407883-supplementary.pdf]

Supplementary material for

Brain Structural Alterations Underlying Mood-Related Deficits in Schizophrenia

Margherita Biondi [1], Marco Marino [2,3], Dante Mantini [3] and Chiara Spironelli [1,2,*]

Between-group, post-hoc analysis on ROI-GMV (*q* threshold set at 0.01) for SZ patients *vs.* healthy controls.

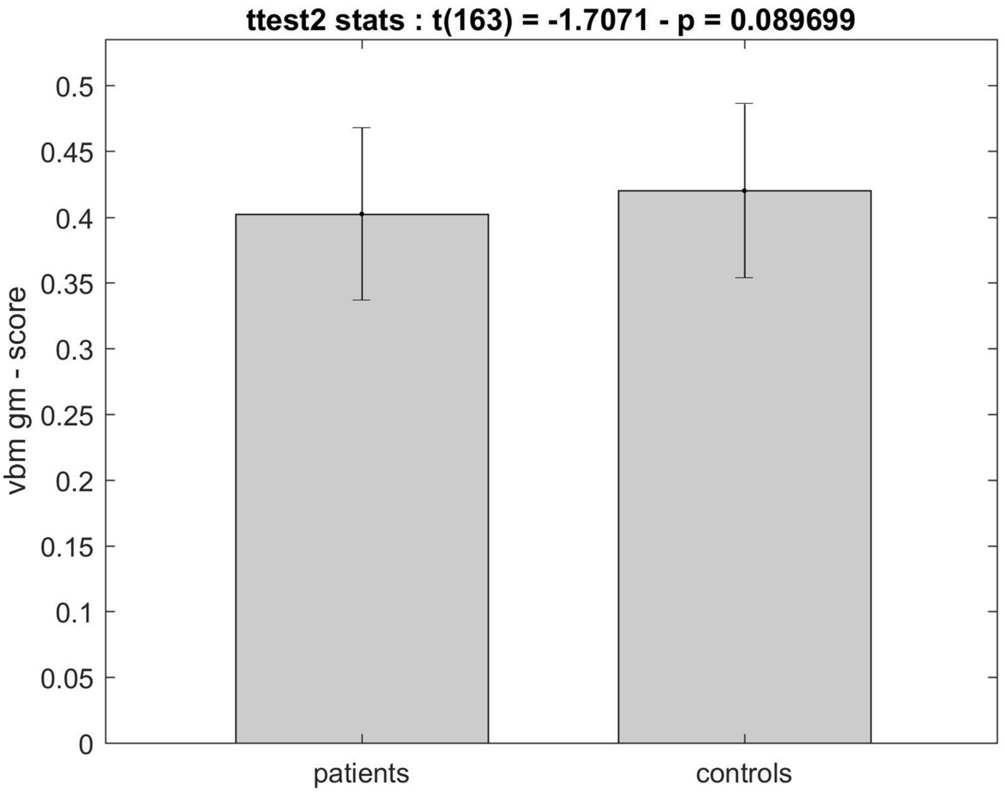

Figure S1. The ROI-based GMV post hoc analysis revealed that, compared with 91 HC participants, the amount of 74 SZ patients' VBM-GMV at the ROI level tended to be reduced ($q < 0.01$).
